# Dry Film Photoresist-Based Microfabrication: A New Method to Fabricate Millimeter-Wave Waveguide Components

**DOI:** 10.3390/mi12030260

**Published:** 2021-03-03

**Authors:** Sadia Farjana, Mohamadamir Ghaderi, Sofia Rahiminejad, Sjoerd Haasl, Peter Enoksson

**Affiliations:** 1 Department of Microtechnology and Nanoscience, Chalmers University of Technology, 41258 Göteborg, Sweden; mohammadamir.ghaderi@chalmers.se (M.G.); sofia.rahiminejad@gmail.com (S.R.); peter.enoksson@chalmers.se (P.E.); 2 Nasa Jet Propulsion Laboratory, Pasadena, CA 91109, USA; 3 RISE Research Institutes of Sweden AB, 16440 Stockholm, Sweden; sjoerd.haasl@ri.se; 4 Enoaviatech AB, 11226 Stockholm, Sweden

**Keywords:** dry film photoresist, gap waveguide, microfabrication, MEMS, THz

## Abstract

This paper presents a novel fabrication method based on dry film photoresists to realize waveguides and waveguide-based passive components operating at the millimeter-wave frequency (30–300 GHz). We demonstrate that the proposed fabrication method has a high potential as an alternative to other microfabrication technologies, such as silicon-based and SU8-based micromachining for realizing millimeter-wave waveguide components. Along with the nearly identical transfer of geometrical structures, the dry film photoresist offers other advantages such as fewer processing steps, lower production cost, and shorter prototyping time over the conventional micromachining technologies. To demonstrate the feasibility of the fabrication process, we use SUEX dry film to fabricate a ridge gap waveguide resonator. The resonator is designed to exhibit two resonances at 234.6 and 284 GHz. The measured attenuation at 234 GHz is 0.032 dB/mm and at 283 GHz is 0.033 dB/mm for the fabricated prototype. A comparative study among different existing technologies indicates that the reported method can give a better unloaded *Q*-value than other conventional processes. The measured unloaded *Q*-values are in good agreement with the simulated unloaded *Q*-values. The signal attenuation indicates that SUEX dry film photoresists can be used to fabricate passive devices operating at millimeter-wave frequencies. Moreover, this new fabrication method can offer fast and low-cost prototyping.

## 1. Introduction

The millimeter-wave (mm-wave) frequency band has gained a lot of attention over the past few years due to growing commercial applications such as automotive radars, security, imaging, and point-to-point communication links [1,2,3,4]. At the mm-wave frequency range, computer numerical control (CNC) milling is still the most common method to manufacture passive components such as waveguides, antennas, and filters. As the operating frequency approaches higher frequencies, the dimension of the waveguide and waveguide-based components continue to decrease. The decreasing feature size of these waveguide components makes the traditional fabrication method more and more challenging in terms of time and cost. A new fast and low-cost manufacturing process to cope with the strict manufacturing tolerances is needed. Micromachining due to the high dimensional accuracy and the possibility of achieving high-aspect-ratio structures has the potential for the manufacturing of mm-wave components. Among different micromachining techniques, silicon-based micromachining [5,6], SU8 photoresist-based process [7,8,9,10,11], and LIGA-based thick layer electroplating [12,13] are suitable for manufacturing mm-wave components.

All the above-mentioned micromachining techniques have both benefits and drawbacks. In silicon micromachining, silicon can be removed by laser micromachining or using deep reactive ion etching (DRIE). Even though DRIE is a well-established process and widely used to fabricate waveguide components above 100 GHz, there are several issues with this process. Si processing requires several processing steps as well as sophisticated fabrication tools. Moreover, it is difficult to achieve vertical sidewalls in the devices which are critical for the high-quality performance of the waveguide devices [14,15,16]. Considering the feature size required in mm-wave applications the DRIE process becomes time intense. Also, critical features like structure with multiple steps, overhang feature are difficult to achieve by this process. Furthermore, it requires cleanroom facilities and special expertise to run the process which increases the production cost. The LIGA process, on the other hand, can produce a high-definition metal structure, but this process suffers from nonuniformity issues and thus needs additional processing steps [17]. Among different photoresists, SU8 is widely used for obtaining structures with high aspect ratios as it can maintain straight sidewalls. Additionally, thicker structures can be achieved simply by optimizing the spinning speed or by multiple spin coatings of the photoresist. However, the fabrication of mm-wave components using SU8-based processes faces several challenges. Achieving the required uniformity using spin coating is often difficult—the layers suffer from edge bead. The reported edge bead for thick SU8 photoresists is higher than 30 µm for a thickness of 100 µm [18]. Furthermore, the thick SU8 layer needs a very long curing time, and is prone to instability and delamination [19,20]. Additionally, the processing time is very long as SU8 needs long relaxation times in between each step to reduce stress. As discussed, the existing fabrication technologies for mm-wave components are either costly, complicated, time-consuming, have some fabrication limitations, or suffers from all the above-mentioned issues. Therefore, there is a need for a new fabrication method that can overcome the challenges of the existing fabrication methods. 

Dry film photoresists are widely used in printed circuit board (PCB) technology [21] and offer many advantages such as no edge bead, thickness uniformity over the whole wafer, fast processing, straight sidewalls, requiring fewer sophisticated tools, and overall lower fabrication cost [22]. Dry film photoresists have already been used to define microfluidic channels [23], electroplate molds for LIGA processes [22,24,25], and for sealing of fluidic channels [26]. Among different dry film photoresists, the epoxy-based dry film photoresist SUEX also appears to be a promising material for a wide range of MEMS applications [27]. However, dry film photoresists have never been used for the fabrication of the waveguide components for radio frequency (RF) applications.

**Figure 1:**
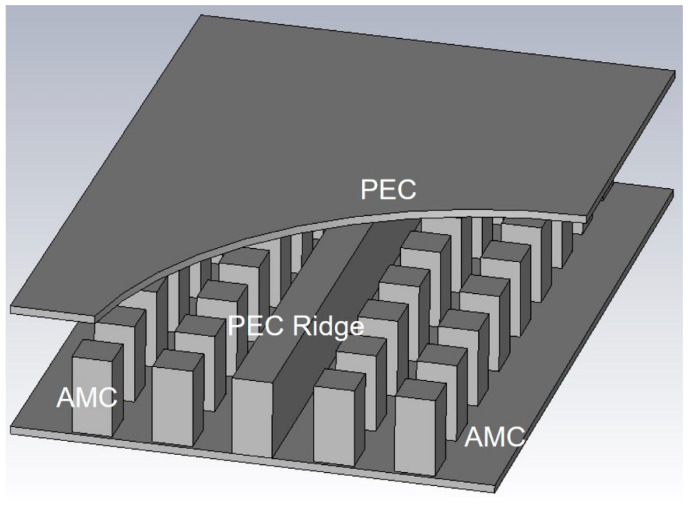
Illustration of a ridge gap waveguide. The ridge and the lid are perfect electric conductor (PEC) surfaces and the pin surface realizes the artificial magnetic conductor (AMC) surface.

On the other hand, gap waveguide technology has gained lots of attention at mm-wave frequencies due to its simple geometry and high fabrication tolerance. The gap-waveguide concept is based on controlling the propagation of the electromagnetic waves in desired directions inside a parallel-plate geometry by using the fundamentals of boundary conditions [28]. When a perfect electric conductor (PEC) and an artificial magnetic conductor (AMC) are placed parallel to each other at a gap smaller than λ/4, no wave can propagate between them [28]. However, if a PEC strip is placed in between the AMC plates, the wave can propagate along the metal strip without leaking away from the structure [29,30,31]. A simple ridge gap waveguide structure is shown in Figure 1. Recently, many gap waveguide passive components have been demonstrated and realized using the conventional manufacturing method [32,33,34] as well as by using micromachining techniques [19,35,36]. 

Once a new fabrication method is proposed and demonstrated, it is important to characterize the electrical performance of the fabricated device. For mm-wave components, loss or attenuation in the waveguide is the key factor that determines the performance of the waveguide-based components. Therefore, we aimed to characterize the loss study of the fabricated device in this study. Losses in the passive waveguide section can be accurately predicted from the quality factor (Q) of a resonator designed in the same specific waveguide technology. As the resonator is very sensitive to fabrication tolerances, the measurement result of a resonator helps to understand the feasibility and viability of the fabrication process.

Previously, the ridge gap resonator was fabricated using silicon micromachining [36], SU8 micromachining [19], carbon nanotubes (CNTs) [37], and off-stoichiometry thiol–enes-epoxy polymer (OSTEMER) [38]. In this work, we demonstrate the application of SUEX dry film photoresist for mm-wave components by fabrication of a ridge gap waveguide resonator working above 200 GHz and analyzing the quality factor of the fabricated resonator. A silicon wafer was used as a carrier and was coated with a SUEX base layer. The measured quality factor (Q-factor) and attenuation demonstrate that SUEX dry film is a suitable material for the fabrication of next-generation mm-wave waveguide components such as antennas, filters, etc. To our knowledge, this is the first dry film photoresist-based gap waveguide resonators working above 200 GHz. This new material-based fabrication method successfully demonstrates that this novel process is compatible with other existing technologies. Moreover, along with delivering a high-aspect-ratio structure with a vertical sidewall, this method reduces the fabrication time and fabrication complexities.

## 2. Structure and Design of Ridge Gap Waveguide Resonator

An open-ended ridge gap waveguide resonator has been designed with one row of pins at the end of the ridge section. The layout of the resonator is similar to the ridge gap resonator for 220–325 GHz presented in [19,36,37,38]. We optimized the design of the ridge gap resonator used in our study based on the thickness of dry films that were available. This is the main reason that the resonator designed for SUEX dry film is not identical to the previously demonstrated ridge gap waveguide resonator [19,36,37,38]. 

**Figure 2:**
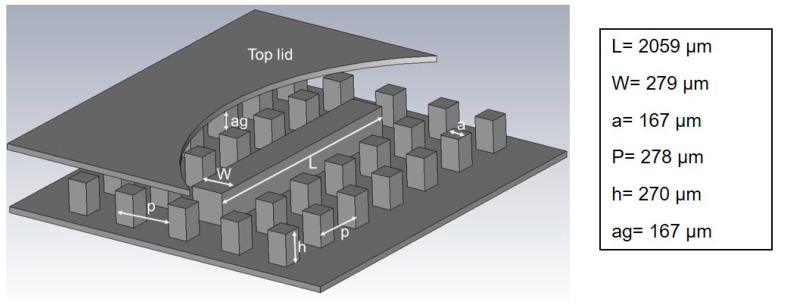
Perspective view of the ridge gap resonator, displaying the dimensions and placements of the pin and the ridge.

To determine the losses in the waveguide, the quality factor (Q-factor) of the resonator was used. To calculate the unloaded Q-factor, a weak coupling must be established between the signal source and the designed resonator. Considering the available thickness of SUEX dry film sheets and to facilitate the fabrication process, the height of the pins and the ridge was optimized. The AMC surface was achieved by square pins of 270 µm height (Figure 2). The dimension of the pins is 167 × 167 µm. The airgap between the AMC and the PEC surface is 167 µm. The height of the ridge section is similar to that of the pins. To obtain weak coupling with the measurement flange transitions, one pin row was added between the waveguide port and the ridge at each side.

**Figure 3:**
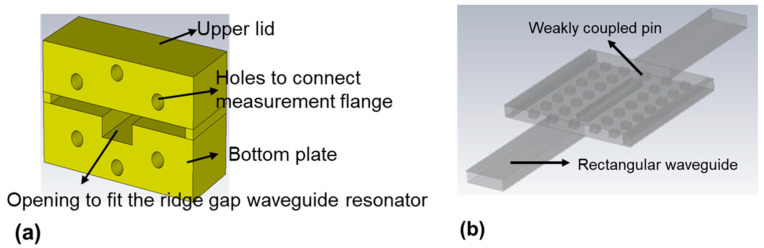
(**a**) Schematic of the support package used during measurements with the PEC lid above; (**b**) schematic of the ridge gap waveguide resonator with waveguide transitions.

A milled support package was used to measure the gold coated SUEX chip. This metal support package was made of aluminum (Al) and provided the lid (PEC) above the active area of the chip to maintain a fixed airgap so that the wave can only propagate along the ridge. The waveguide flanges were connected directly to the resonator, through the holes made in the external package (Figure 3). A schematic of the support package and the ridge gap waveguide resonator with waveguide transitions is shown in Figure 3.

## 3. Materials and Method

### 3.1. Materials

A 6-inch silicon wafer was used as a substrate. SUEX epoxy dry film (DJ MICROLAMINATES Inc., Berlin, Germany) sheets of 6-inch wafer cut sizes and different thicknesses were used to obtain the device structure. For the base layer, a 40 µm SUEX dry film sheet was used. SUEX dry film sheets of 200, 50, and 20 µm were used to obtain the structure layer. The developer mr-DEV 600 (microresist technology) was used to develop the structure. The device was made conducive by sputtering titanium (Ti) and gold (Au).

### 3.2. Dry Film Lamination Method

**Figure 4:**
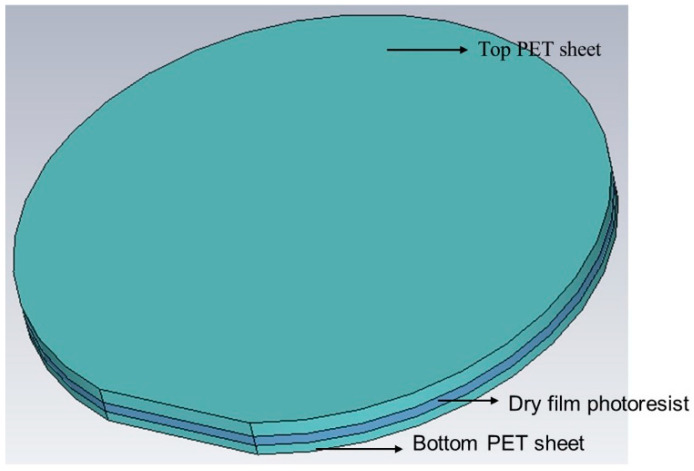
Structure of the dry film photoresist.

A commercial laminator (PRO SERIESTM 3600) was used to laminate SUEX dry film sheets. SUEX dry film sheets were sandwiched between two polyester (PET) films. Figure 4 is the schematic of a dry film sheet covered with a PET sheet on each side. The PET sheet of one side was removed before laminating the photoresist against the substrate. The lamination of the resist was carried out at controlled temperature and pressure. The lamination temperature of the roller depended on the thickness of the dry film sheet. The PET sheet that was kept during lamination needed to be removed before exposing the laminated layer or laminating another dry film sheet. 

### 3.3. Fabrication Method

**Figure 5:**
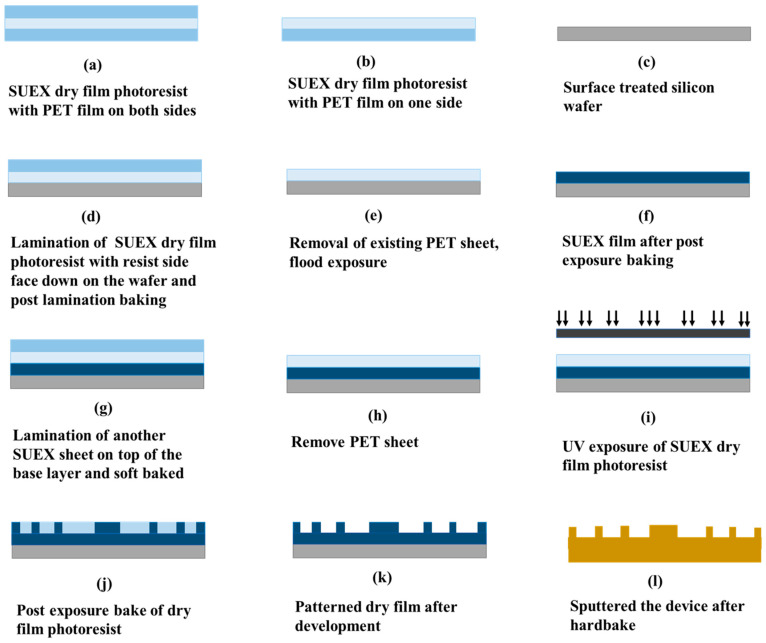
Schematic of the complete fabrication process. (**a**) SUEX dry film photoresist with polyester (PET) film on both sides; (**b**) removing the PET from one side; (**c**) O_2_ plasma treatment of the silicon wafer; (**d**) lamination of SUEX dry film photoresist with resist side face down on the wafer and post lamination baking; (**e**) removal of existing PET sheet and flood exposure; (**f**) SUEX film after postexposure baking; (**g**) lamination of another SUEX sheet on top of the base layer and soft baking; (**h**) removal of PET sheet; (**i**) Patterning of SUEX dry film photoresist; (**j**) postexposure bake of dry film photoresist; (**k**) patterned dry film after development; (**l**) sputtering of the final structure.

To demonstrate the SUEX dry film-based resonator, a silicon wafer was used as a substrate. A 40 µm SUEX layer was laminated on the silicon wafer as a base layer. The final structure was built on this polymer layer. The reason to use a base layer was to obtain a complete polymer structure. The height of the pins and ridge of the resonator are 270 µm. To obtain a 270 µm thick resist layer, we laminated three SUEX dry film sheets of 200, 50, and 20 µm. The complete fabrication process is described below and shown in Figure 5.

#### 3.3.1. Fabrication of the Base Layer

Before laminating the SUEX dry film sheets, the wafer needs to be treated for better adhesion. Plasma cleaning was carried out followed by a dehydration bake at 200 °C for 15 min. To obtain a polymer base layer, we laminated a 40 µm SUEX dry film sheet on the silicon wafer. The laminator temperature was set to 65 °C and the speed of the lamination was maintained at 5 mm/sec. A post lamination bake at 80 °C for 1 min was carried out on a hot plate for better adhesion with the silicon wafer. After post lamination bake, the remaining PET sheet was removed, and the laminated SUEX layer was exposed to an energy of 1200 mJ/cm^2^. A postexposure bake at 95 °C for 10 min was carried out followed by an oxygen plasma ashing before laminating the next SUEX sheet.

#### 3.3.2. Fabrication of the Device Layer

As mentioned earlier, the heights of the resonator pins and the ridge were designed to be 270 µm. Three SUEX dry film sheets were used to obtain the required photoresist thickness. After removing the PET sheet of a 200 µm SUEX sheet, the SUEX film was laminated on the base layer. Laminator temperature and speed were 65 °C and 5 mm/sec, respectively. A post lamination bake at 80 °C for 1 min 30 sec was carried out on a hot plate to ensure adhesion of the laminated layer with the base photoresist layer. The PET sheet was removed after the post lamination bake and a 50 µm SUEX sheet was laminated. Lamination temperature and speed were kept the same as those for the 200 µm SUEX sheet. After this, a post lamination bake was carried out at 80 °C for 1 min. Finally, a 20 µm SUEX dry film sheet was laminated at 62 °C and a speed of 5 mm/sec followed by a post lamination bake for 1 min at 80 °C. Once all the layers were laminated, the sheets were exposed to UV with a mask to obtain the patterns of the device. SUEX is a negative photoresist and the mask was designed considering this. The exposure energy of 9000 mJ/cm^2^ was used to crosslink the 270 µm thick SUEX film. A postexposure bake was carried out in two steps. The baking started at 65 °C on a hot plate for 10 min followed by a bake at 95 °C for 1 h. 

Finally, the whole wafer was developed in mr-DEV 600 for 30 min with no agitation. The development was carried out in two different baths. After 20 min, the first bath was changed, and the wafer was moved to a new container with the fresh developer and kept there for 10 min. Once the development was over, the wafer was rinsed with an IPA solution and kept in an IPA bath for 5 min. The drying process was carried out on a hot plate at 100 °C. The wafer was placed on a hot plate right after removing it from the IPA solution. The drying process was continued for 5 min followed by a hard bake at 150 °C for 10 min. The wafer was then diced into pieces. To make the chip conductive, Au was used, and we sputtered both sides of the structure with 50 nm Ti and 900 nm Au.

## 4. Result and Discussion

A ridge gap resonator has been used to demonstrate the application of SUEX dry film photoresist for the fabrication of mm-wave waveguide and passive waveguide components. In this section, we present the fabrication result, measurement result and a comparative study among different microfabrication methods.

### 4.1. Fabrication Result

**Figure 6:**
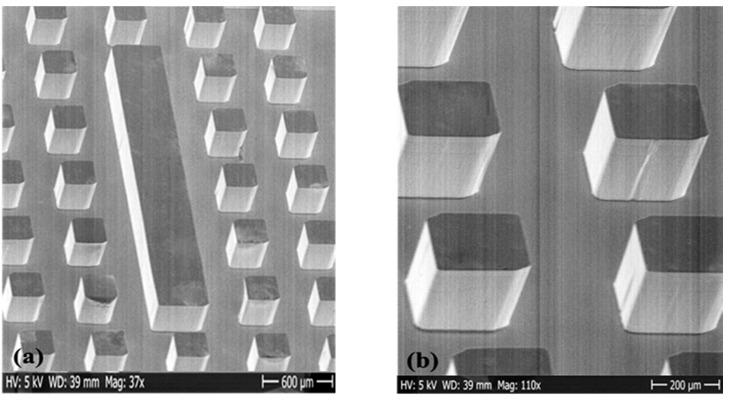
(**a**) SEM image of SUEX ridge gap resonator with a pin height of 270 µm; (**b**) closed view of the pins of SUEX ridge gap resonator.

SEM image of a fabricated SUEX ridge gap waveguide resonator is shown in Figure 6. The SEM image shows that the sidewalls have a high verticality. The height of the realized structure was measured to be 272 µm ± 2 µm which, compared to the target thickness of 270 µm, exhibited an excellent thickness uniformity and flatness over the laminated area. Measured surface roughness of the fabricated device was 3.81 ± 0.5 nm.

### 4.2. Measurement Result

**Figure 7:**
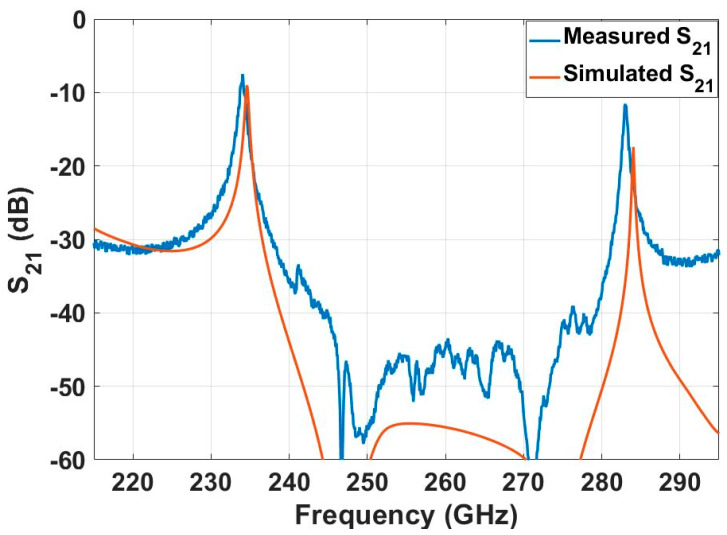
Full electromagnetic wave simulation compared with measurement results between 220 and 320 GHz. The two resonant frequencies are around 234 and 284 GHz.

**Figure 8:**
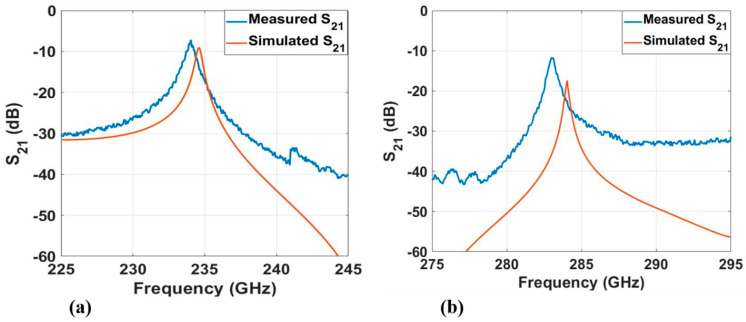
Measured and simulated S_21_ of the dry film resonator (**a**) at 215–245 GHz, showing a resonance peak at around 234 GHz, and (**b**) at 275–295 GHz, showing a resonance peak at around 184 GHz.

Two-port S-parameter measurements were performed for the fabricated resonator. S_21_ was measured using a Keysight PNA-X network analyzer and VDI WR 3.4 frequency extender modules. Two measurements were performed. The first measurement was carried out with a broadband sweep. The result is shown in Figure 7. However, narrowband measurements were also carried out in the vicinity of the resonance peak at 215–245 GHz and 275–295 GHz. Figure 8 present the measured and simulated S_21_ of the dry film resonator at frequency ranges 215–245 GHz and 275–295 GHz, respectively.

Table 1 presents the unloaded *Q*-values and loss of the dry film resonator. The unloaded Q-factor of the resonator was obtained by using the following equations [39,40]

(1) $$\frac{1}{Q_{L}}=\frac{1}{Q_{U}}+\frac{1}{Q_{E}}$$

(2) $$Q_{U}=\frac{Q_{L}}{1-S_{21}}$$

(3) $$Q_{L}=\frac{f_{o}}{\Delta f_{3dB}}$$

(4) $$Q_{E}=10^{-\left[S_{21}\left(dB\right)/20\right]}\;\cdot Q_{L}$$

where *Q_L_* is the loaded Q-factor, *Q_U_* is the unloaded Q-factor, *Q_E_* is the external Q-factor (due to the loading effect), *f_o_* is the resonance frequency, and Δ*f*_3*dB*_ is the 3-dB bandwidth of the resonance. The attenuation constant can also be extracted from the unloaded *Q*-values by using Equation (5).

(5) $$\alpha =\;\frac{\beta }{2Q_{U}}$$

where *α* = attenuation constant and *β* = propagating constant.

### 4.3. Comparative Study with the Existing Technologies

In the introduction, we mentioned different fabrication techniques to manufacture mm-wave waveguides and passive waveguide components and the benefits and drawbacks of those processes. Although silicon and SU8 photoresist processes are the most commonly used micromachine techniques to fabricate waveguide components for mm-waves, other microfabrication techniques have also been examined for fabricating mm-wave gap waveguide components [19,35,36,37,38]. The ridge gap resonator was fabricated by using silicon micromachining [36], SU8 micromachining [19], carbon nanotubes (CNTs) [37], and an off-stoichiometry thiol-enes-epoxy polymer (OSTEMER) [38]. In all the above-mentioned processes, a conductive layer was deposited to achieve an electrically conductive structure. There are many reasons to use different materials—one is to find a suitable material and method that are appropriate for batch fabrication in a simplified way and in a shorter time, with a low production cost. The main difference among the processes was the fabrication steps involved to obtain the required structure. In this section, we will present a performance comparison analysis among different fabrication methods used to fabricated ridge gap resonator to understand the feasibility of the processes to fabricate mm-wave waveguide components. Additionally, we will reflect on the processing time and processing cost utilized by these processes and the convenience of the methods to be used for batch fabrication of mm-wave components. 

Silicon-based fabrication of ridge gap resonators relies on patterning and deep etching of bulk silicon wafer [36]. The etch rate of silicon is comparatively low, about 1–5 µm/min. Furthermore, the sidewall angle often depends on the size of the etching area, thus achieving straight sidewalls requires significant parameter optimization. Finally, the height of structures is usually controlled only by the etching time which limits the height accuracy of the realized structures. Although the measured unloaded *Q*-value of the silicon ridge gap resonator showed good agreement with the simulated unloaded *Q*-value, the processing time and complexity were very high. Considering the device performance, the Si process is an established process, but it is not a suitable process for batch fabrication of mm-wave components as it is a time-consuming and complex process.

A SU8 ridge gap resonator was made of a two-layer SU8 and was presented in [19]. The SU8 based resonator involved process steps such as resist spin coat, soft bake, exposure, postexposure bake for each layer, and development. By optimizing the spin speed, a very thick SU8 layer is possible to achieve. The soft bake time usually required to obtain structures of 277 ± 10 µm height was approximately 90 to 120 min [35]. Additionally, a significant amount of relaxation time between each step is required which made the SU8-based processing very prolonged. Even though the processing time of the SU8 process was similar to that of a silicon process, the SU8 processing is generally less complex and requires fewer optimization steps. The fabricated structures, however, show negative sidewall angles, resulting in a mushroom-like structures. The measured loss at 234 GHz was almost double than that of the silicon ridge gap resonator. The measured loss at 284 GHz on the other hand was similar to that of the silicon ridge gap resonator. Nonuniform resist distribution could be a reason for height variation among the structures of the device and that could be the reason for higher loss at 234 GHz.

To fabricate a CNT-based ridge gap resonator, processing steps of lithography and growth of CNT by using thermal chemical vapor deposition (CVD) were utilized. The CNT-based fabrication process was faster compared with silicon micromachining but the reported loss for CNT-based ridge gap resonator was significantly higher compared to that of the silicon-based ridge gap waveguide resonator [37]. Thus, CNTs are challenging to use in the fabrication of mm-wave waveguide components.

A ridge gap waveguide resonator made of OSTE (litho) polymer was reported in [38]. The OSTE-based fabrication process involves fewer processing steps and is shorter compared to other fabrication techniques but suffers from flatness issues. Additionally, the measured shift in the resonance frequency indicates that this is not a suitable process and requires a significant amount of work and tuning of the process to be used in the future. Additionally, OSTE (litho) is not able to deliver critical features such as overhang or multiple height structures that is generally needed for mm-wave waveguide applications. 

Additionally, many gap waveguide-based passive components have been fabricated by using the CNC milling technique. The tolerance required by CNC milling at the mm-wave frequency range is quite high. Specially manufacturing waveguide components operating above 100 GHz is challenging, time-consuming, and expensive for the CNC milling technique as a very small milling tool is used.

On the other hand, the dry film photoresist not only delivers better structures with better fabrication tolerances, but it also reduces the processing time as well as the production cost. Furthermore, the tools used for this process are readily available and low-cost. Therefore, the overall fabrication cost decreases compared to the conventional fabrication techniques. Moreover, the measurement result and the possibility of batch fabrication indicates that this processing will be a suitable method for manufacturing of waveguide components operating above 100 GHz.

Table 2 presents a performance comparison of the unloaded *Q*-values and losses from simulations and previous silicon, SU8, CNT, and OSTE devices of identical design to the ridge gap resonator fabricated with SUEX dry film photoresist. Table 3 shows that the dry film-based method delivers the best result among the above-discussed fabrication methods. Structures obtained by SUEX dry film have high accuracy in terms of thickness uniformity, flatness, and reliable transfer of the layout resulting in an improved device performance.

As mentioned in the introduction, for mm-wave components, loss, or attenuation in the waveguide is the key factor that determines the performance of the waveguide-based components. Factors that affect the attenuation in a waveguide are conductivity of the walls, and the dielectric inside the waveguide. As gap waveguide is free from any dielectric loss, the main factor contributing to the attenuation is the conductivity of the walls. Measured surface roughness of the fabricated device is 3.81 ± 0.5 nm. The sidewall angle of the large aspect ratio components is an important structural factor. This is a main concern in DRIE-based processes in silicon technology where achieving a vertical sidewall is challenging. Furthermore, the process often suffers from scalloping on the sidewalls. However, as proven by SEM images, the dry film technology can achieve smooth and near-vertical sidewalls which is crucial for electromagnetic applications. The measured *Q*-value presented in Table 1 show good agreement with the measured *Q*-value for the resonator, which has a good agreement with the simulated *Q*-value.

Table 3 shows a comparison table of different microfabrication techniques used to fabricate mm-wave waveguide components. Comparisons were carried out based on processing steps, lab facilities required by the technologies, processing time, and processing cost. While comparing different fabrication methods, we also considered their ability to deliver critical features such as overhang features, structures containing multiple heights/steps, and device performance targeting mm-wave applications.

## 5. Conclusions 

We presented a straightforward fabrication method to fabricate mm-wave waveguide and passive waveguide components. To realize the feasibility of the fabrication method, a ridge gap waveguide resonator was demonstrated. This paper aims to demonstrate the use of dry film as an alternative to conventional DRIE and liquid SU8 photoresists for the fabrication of mm-wave waveguide components. Along with delivering geometrical structures with high accuracy, dry film photoresists offer other advantages such as fewer processing steps, shorter prototyping time, and low processing cost as the production time is reduced. The measured attenuation at 234 GHz is 0.032 dB/mm and at 283 GHz is 0.033 dB/mm, which shows a good agreement with the unloaded *Q*-value obtained from the simulation and shows better values than previously presented methods.

## Figures and Tables

**Table 1:** Simulated and measured unloaded quality (*Q_u_*)-values and loss/mm.

Parameters	Simulated		Measured	
Frequency (GHz)	234.6	284	234	283
*Q_u_*-value	802	938	656	786
Loss (dB/mm)	0.026	0.028	0.032	0.033

**Table 2:** Performance comparison of the unloaded *Q*-values and losses from simulations, and previous Si, SU8, carbon nanotube (CNT), and off-stoichiometry thiol–enes-epoxy (OSTE) devices of identical design with the ridge gap resonator fabricated by the SUEX dry film photoresist.

Fabrication Method	Simulations			Measurement			*f_offset_* (%)	*Q_Uoffset_* (%)
	*f* (GHz)	*Q_U_*	α (dB/m)	*f* (GHz)	*Q_U_*	α (dB/m)		
**First Resonance**								
**Silicon** [36]	234	859	0.025	234	642	0.033	0	25.26
**SU8** [19]	234	859	0.025	233	319	0.067	0.42	62.86
**CNT** [37]	234	859	0.025	236	274	0.079	0.85	68.1
**OSTE(litho)** [38]	234	859	0.025	238	314	0.064	1.71	63.44
**This work**	234.6	802	0.026	234	656	0.032	0.25	18.14
**Second Resonance**								
**Silicon**	284	992	0.026	283	628	0.043	0.35	36.69
**SU8**	284	992	0.026	283	628	0.041	0.35	36.69
**CNT**	284	992	0.026	289	518	0.051	1.76	47.78
**OSTE(litho)**	284	992	0.026	292	210	0.127	2.81	78.83
**This work**	284	938	0.028	283	786	0.033	0.35	20.76

**Table 3:** Comparison based on processing steps, lab facility required by the technologies, processing time, and processing cost of different microfabrication techniques used to fabricate mm-wave waveguide components.

Technology		Silicon	SU8	CNT	OSTE (litho)	Dry Film
**Processing step**	**Surface pretreatment**	Dehydration bake, hard mask deposition	Dehydration bake	Dehydration bake	Antistick layer	Dehydration bake
	**Lithography step**	Process tool *	Process tool **	Process tool *	Process tool ***	Process tool ****
	**Special processing step**	DRIE	none	CVD	Degasification	Lamination
**Processing time**		15 h/wafer	10 h/wafer	10 h/wafer	3 h/wafer	3 h/wafer
**Ability to obtain multiple heights structure**		Difficult	Difficult	Not possible	Not possible	Possible with high accuracy
**Device Performance**		Good	Medium	Not satisfactory	Not satisfactory	Good

* Process tools: spinner, hotplate, mask aligner/laser writer, development bath, liftoff bath; ** process tools: spinner, hotplate, mask aligner, development bath; *** process tools : hotplate, mask aligner, development bath; **** process tools: hotplate, mask aligner, development bath; processing time: The processing times are indicative of the design and can vary depending on the tools and specifics of the design.

## References

[1] Hasch J., Topak E., Schnabel R., Zwick T., Weigel R., Waldschmidt C. (2012). Millimeter-Wave Technology for Automotive Radar Sensors in the 77 GHz Frequency Band. IEEE Trans. Microw. Theory Tech..

[2] Yang X., Liu L., Vaidya N.H., Zhao F. A vehicle-to-vehicle communication protocol for cooperative collision warning. Proceedings of the First Annual International Conference on Mobile and Ubiquitous Systems: Networking and Services, 2004. MOBIQUITOUS 2004.

[3] Smulders P.P. (2002). Exploiting the 60 GHz band for local wireless multimedia access: Prospects and future directions. IEEE Commun. Mag..

[4] Hansen C.J. (2011). WiGiG: Multi-gigabit wireless communications in the 60 GHz band. IEEE Wirel. Commun..

[5] Blondy P., Brown A.R., Crost D., Rebeiz G.M. Low loss micromachined filters for millimeter-wave telecommunication systems. Proceedings of the 1998 IEEE MTT-S International Microwave Symposium Digest (Cat. No.98CH36192).

[6] Li Y., Kirby P.L., Papapolymerou J. Silicon Micromachined W-Band Folded and Straight Waveguides Using DRIE Technique. Proceedings of the 2006 IEEE MTT-S International Microwave Symposium Digest.

[7] Tian Y., Shang X., Wang Y., Lancaster M.J. (2015). Investigation of SU8 as a structural material for fabricating passive millimeter-wave and terahertz components. J. Micro/Nanolithogr. MEMS MOEMS.

[8] Wang Y., Ke M., Lancaster M.J., Chen J. (2011). Micromachined 300-GHz SU-8-Based Slotted Waveguide Antenna. IEEE Antennas Wirel. Propag. Lett..

[9] Lorenz H., Despont M., Fahrni N., Labianca N.C., Renaud P., Vettiger P. (1997). SU-8: A low-cost negative resist for MEMS. J. Micromech. Microeng..

[10] Smith C.H., Barker N.S. SU-8 micromachining process for millimeter and submillimeter-wave waveguide circuit fabrication. Proceedings of the 2008 33rd International Conference on Infrared, Millimeter and Terahertz Waves.

[11] Iii C.H., Xu H., Barker N. Development of a multi-layer SU-8 process for terahertz frequency waveguide blocks. Proceedings of the Microwave Symposium Digest, 2005 IEEE MTT-S International.

[12] Liao Y.-S., Chen Y.-T. (2005). Precision fabrication of an arrayed micro metal probe by the laser-LIGA process. J. Micromech. Microeng..

[13] Arnaudov R., Avdjiiski B., Kostov A., Videkov V., Andreev S., Yordanov N. (2006). Novel Microcontacts in Microwave Chip Carriers Developed by UV-LIGA Process. IEEE Trans. Adv. Packag..

[14] Kirby P., Pukala D., Manohara H., Mehdi I., Papapolymerou J. (2006). Characterization of micromachined silicon rectangular waveguide at 400 GHz. IEEE Microw. Wirel. Compon. Lett..

[15] Chattopadhyay G., Ward J.S., Manohara H., Toda R. Deep Reactive Ion Etching based silicon micromachined components at terahertz frequencies for space applications. Proceedings of the 2008 33rd International Conference on Infrared, Millimeter and Terahertz Waves.

[16] Leong K.M.K.H., Hennig K., Zhang C., Elmadjian R.N., Zhou Z., Gorospe B.S., Chang-Chien P.P., Radisic V., Deal W.R. (2012). WR1.5 Silicon Micromachined Waveguide Components and Active Circuit Integration Methodology. IEEE Trans. Microw. Theory Tech..

[17] Nordquist C.D., Wanke M.C., Rowen A.M., Arrington C.L., Lee M., Grine A.D. Design, fabrication, and characterization of metal micromachined rectangular waveguides at 3 THz. Proceedings of the 2008 IEEE Antennas and Propagation Society International Symposium.

[18] Abada S., Salvi L., Courson R., Daran E., Reig B., Doucet J.B., Camps T., Bardinal V. (2017). Comparative study of soft thermal printing and lamination of dry thick photoresist films for the uniform fabrication of polymer MOEMS on small-sized samples. J. Micromech. Microeng..

[19] Rahiminejad S., Pucci E., Haasl S., Enoksson P. (2014). SU8 ridge-gap waveguide resonator. Int. J. Microw. Wirel. Technol..

[20] Mata A., Fleischman A.J., Roy S. (2006). Fabrication of multi-layer SU-8 microstructures. J. Micromech. Microeng..

[21] Vollenbroek F.A., Spiertz E.J. (1988). Photoresist systems for microlithography. Electronic Applications.

[22] Kukharenka E., Farooqui M.M., Grigore L., Kraft M., Hollinshead N. (2003). Electroplating moulds using dry film thick negative photoresist. J. Micromech. Microeng..

[23] Vulto P., Glade N., Altomare L., Bablet J., Del Tin L., Medoro G., Chartier I., Manaresi N., Tartagni M., Guerrieri R. (2005). Microfluidic channel fabrication in dry film resist for production and prototyping of hybrid chips. Lab Chip.

[24] Ehrfeld W., Hessel V., Lowe H., Schulz C., Weber L. (1999). Materials of LIGA technology. Microsyst. Technol..

[25] Lorenz H., Paratte L., Luthier R., De Rooij N.F., Renaud P. (1996). Low-cost technology for multilayer electroplated parts using laminated dry film resist. Sens. Actuators A Phys..

[26] Chartier I., Sudor J., Fouillet Y., Sarrut N., Bory C., Gruss A. (2003). Fabrication of a Hybrid Plastic-Silicon Microfluidic Device for High-Throughput Genotyping.

[27] Johnsona D.W., Goettertb J., Singhb V., Yemaneb D. (2012). SUEX Dry Film Resist@—A new Material for High Aspect Ratio Lithography. Mater. Sci..

[28] Rajo-Iglesias E., Kildal P.-S. (2011). Numerical studies of bandwidth of parallel-plate cut-off realised by a bed of nails, corrugations and mushroom-type electromagnetic bandgap for use in gap waveguides. IET Microw. Antennas Propag..

[29] Kildal P. Three metamaterial-based gap waveguides between parallel metal plates for mm/submm waves. Proceedings of the 2009 3rd European Conference on Antennas and Propagation.

[30] Valero-Nogueira A., Alfonso E., Herranz J., Kildal P.-S. (2009). Experimental Demonstration of Local Quasi-TEM Gap Modes in Single-Hard-Wall Waveguides. IEEE Microw. Wirel. Compon. Lett..

[31] Valero-Nogueira A., Baquero M., Herranz-herruzo J., Domenech J., Alos E.A., Vila A. (2011). Gap Waveguides Using a Suspended Strip on a Bed of Nails. IEEE Antennas Wirel. Propag. Lett..

[32] Liu J., Vosoogh A., Zaman A.U., Yang J. (2018). A Slot Array Antenna with Single-Layered Corporate-Feed Based on Ridge Gap Waveguide in the 60 GHz Band. IEEE Trans. Antennas Propag..

[33] Vosoogh A., Sorkherizi M.S., Vassilev V., Zaman A.U., He Z.S., Yang J., Kishk A.A., Zirath H. (2019). Compact Integrated Full-Duplex Gap Waveguide-Based Radio Front End For Multi-Gbit/s Point-to-Point Backhaul Links at E-Band. IEEE Trans. Microw. Theory Tech..

[34] Vosoogh A., Haddadi A., Zaman A.U., Yang J., Zirath H., Kishk A.A. (2018). W-Band Low-Profile Monopulse Slot Array Antenna Based on Gap Waveguide Corporate-Feed Network. IEEE Trans. Antennas Propag..

[35] Farjana S., Rahiminejad S., Zaman A.U., Hansson J., Ghaderi M.A., Haasl S., Enoksson P. Polymer based 140 GHz Planar Gap Waveguide Array Antenna for Line of Sight (LOS) MIMO Backhaul Links. Proceedings of the 2019 IEEE MTT-S International Microwave Workshop Series on Advanced Materials and Processes for RF and THz Applications (IMWS-AMP).

[36] Rahiminejad S., Zaman A., Pucci E., Raza H., Vassilev V., Haasl S., Lundgren P., Kildal P.-S., Enoksson P. (2012). Micromachined ridge gap waveguide and resonator for millimeter-wave applications. Sens. Actuators A Phys..

[37] Saleem A., Rahiminejad S., Desmaris V., Enoksson P. (2014). Carbon Nanotubes as Base Material for Fabrication of Gap Waveguide Components. Procedia Eng..

[38] Rahiminejad S., Hansson J., Köhler E., van der Wijngaart W., Haraldsson T., Haasl S., Enoksson P. Rapid manufacturing of OSTE polymer RF-MEMS components. Proceedings of the 2017 IEEE 30th International Conference on Micro Electro Mechanical Systems (MEMS).

[39] Pozar D.M. (2012). Microwave Engineering.

[40] Pucci E., Zaman A.U., Rajo-Iglesias E., Kildal P., Kishk A. (2013). Study of Q -factors of ridge and groove gap waveguide resonators. IET Microw. Antennas Propag..

